# Attitude and knowledge of medical students about organ donation – training needs identified from a Canadian survey

**DOI:** 10.1186/s12909-021-02736-2

**Published:** 2021-07-05

**Authors:** Philippe Robert, Félix Bégin, Sasha Ménard-Castonguay, Anne-Julie Frenette, Hector Quiroz-Martinez, François Lamontagne, Emilie-Prudence Belley-Côté, Frédérick D’Aragon

**Affiliations:** 1 Faculty of Medicine and Health Sciences, Université de Sherbrooke, 3001, 12e Avenue Nord, Sherbrooke, QC J1H 5N4 Canada; 2 Faculty of Medicine, Université Laval, 1050, Avenue de la Médecine, Québec, QC G1V 0A6 Canada; 3 Faculty of Pharmacy, Université de Montréal, 2940, Chemin de Polytechnique, Montréal, QC H3T 1J4 Canada; 4 Faculty of Health Sciences, McMaster University, 1200 Main Street West, Hamilton, ON L8N 3Z5 Canada

**Keywords:** Organ donation, Organ transplantation, Medical education, Medical students, Knowledge, Attitude

## Abstract

**Background:**

Appropriate training of health professionals has been recommended to increase organ donation rates. Some studies have shown insufficient knowledge among medical students. This survey aims to describe their knowledge and attitude toward organ donation (OD).

**Method:**

We designed, pre-tested and conducted an online survey of all undergraduate medical students from Montreal, Laval and Sherbrooke universities in 2016–2017. Multivariate linear regression identified factors associated with a better knowledge score.

**Results:**

Twenty-two percent (750/3344) of students completed the survey. Ninety-one percent of students adequately knew that neurological death is irreversible; 76% acknowledged that someone could be neurologically deceased while his heart is still beating; 69% were not aware of circulatory determination of death. For only one knowledge item, senior students had a better answer than junior students. Total knowledge score was associated with exposure to OD during medical studies and comfort in answering patients’ questions about OD (*p* < 0,001). Regarding attitude, 96% of respondents wished to become organ donors after death and 92% supported OD training during their medical training.

**Conclusion:**

Despite a favourable attitude, medical students have limited knowledge of OD. Findings suggest the need for a formal curriculum about OD, as students expressed.

**Supplementary Information:**

The online version contains supplementary material available at 10.1186/s12909-021-02736-2.

## Introduction

Many people are awaiting a lifesaving organ transplantation: they were 4351 in Canada and 805 in Quebec in 2018 [1]. At the same time, 223 Canadians died awaiting transplantation, including 28 in Quebec [1]. The organ shortage is multifactorial: reasons include a lack of recognition of potential donors, low consent rate, and suboptimal care of potential donors [2, 3]. While the deceased donor rate was 22 per million inhabitants in Canada in 2017, some countries achieve much higher rates, such as 32 in the United States and 47 in Spain [4]. Although these comparisons are limited by methodological differences, Spain is recognized to benefit from a highly coordinated system, with dedicated trained professionals in hospitals [5]. Appropriate training of physicians, nurses and organ donation coordinators can increase consent rate [6–8]. Many health organizations, such as Canadian Blood Services and the National Institute for Health and Care Excellence (NICE) in the United Kingdom, recommend better training of healthcare professionals about organ donation [9, 10]. According to the Medical Council of Canada, graduating medical students should be able to “ensure that appropriate organ donation protocol be activated, in case where brain death has occurred” [11]. It includes to “counsel the family about the possibility [of organ donation]» [11] Inadequate knowledge about organ donation has been shown among health professionals and medical students around the world [12–16] but little data exists about the attitude and knowledge of Canadian medical students. According to a survey conducted in a medical school from Ontario in 2003, students had on average 6.7 correct answers among 14 questions, increasing slightly from the first year (5.7) to the fourth year (7.4) [17]. The number of correct answers was also correlated with previous teaching about organ donation and student comfort with approaching a family about organ donation. For example, 77% of respondents correctly answered the item, “Requesting organ donation usually does not add to the family’s grief”; 64% to “Brain death means that the patient is dead, not in coma”; 50% to “People of all religious groups should be approached about organ donation; 41% to item “Family wishes override wishes expressed on a donor card”; and 32% to “If possible, other family members should be with the next of kin when the request for organ donation is made.” [17] Since medical training and attitude toward organ donation may vary in time and between provinces, this survey aims primarily to describe Quebec undergraduate medical students’ knowledge and the attitude concerning organ donation (OD). Secondarily, it intends to verify whether senior students have better knowledge, and to explore other hypothetical factors. The ultimate goal is to guide future pedagogical interventions in medical education.

## Methods

The survey was developed by a panel of medical students (FB, PR, SMC), experts in organ donation (FDA, AJF, HQM) and experts in methodology (FL, EPBC). We followed a standardized approach to design and conduct a self-administered survey, including items’ generation and reduction, survey validation for clarity, redundancy and comprehensiveness, and test-retest reliability assessment.

### Sampling of survey participants

For this cross-sectional and self-administered survey, the whole population included all medical students in the three French-speaking medical schools in Quebec (i.e.; Laval, Montreal, Sherbrooke). The other medical school in Quebec was not included because of administrative constraints. Each medical students’ association sent the survey to all their members, which correspond to all medical students from these universities.

### Survey development

Items were generated from a literature review and the panel’s hypothesis. Three investigators (PR, FB, SMC) independently search in MEDLINE from inception up to July 2016. The subject headings used were “Tissue and Organ Procurement,” “Organ Transplantation,” “Organ Donation,” combined with the words “Attitude,” “Knowledge,” “Medical Education” and “Medical Student.” The panel generated a list of items in two domains (i.e., knowledge related to OD, attitude about OD). Items were reduced by five members of the panels (AJF, EPBC, FDA, FL, HQM) to select most relevant items to the survey objectives and to limit redundancy. Items assess the four components of the planned behaviour theory, whereby attitude (*toward organ donation and toward the role of the physician in the organ donation system*), subjective norms (*perception of social and professional pressure to participate in organ donation system*) and control perception (*how easy or difficult is to participate in organ donation system*) predict intention (*to participate in the organ donation system*), which in turn predicts behaviour [18, 19]. Comparison of these components and items is provided in Additional file (Table A1). This theory has already been used to explain nurses’ involvement with organ donation [18]. The final survey included 28 questions (9 questions about knowledge and 16 questions about attitude or its potential determinants, plus 3 demographic questions).

### Survey validation

We pre-tested the questionnaire with ten students to assess questionnaire flow, redundant questions, ease of administration, time required and comprehensiveness of the questions. Students took a mean time of 5.3 min to complete the survey (SD 1.8 min). Minor changes were made according to students’ comments. Reliability was assessed for main items through test-retest with the same group of medical students with a three-day interval (Additional file, Table A2).

### Survey administration and ethics

Medical student associations send to their members an email invitation containing a link to the electronic survey, based on Lime Survey™ platform. The survey was accessible for 8 weeks from December 2016 to January 2017 and two email reminders were sent to students. Participation was voluntary and anonymous. Informed consent was obtained from all participants. After having sent the survey, participants could choose to provide their email address in a separate form, to participate in a draw for two $75 gift cards from sports equipment store. This study was approved by the Research Ethics Board of *CIUSSS de l’Estrie-CHUS* (#2017–1588).

### Statistical analysis

Descriptive analyses were performed with SPSS, considering a *P*-value inferior to 0.05 as significant. Knowledge was described with proportions of correct answers (considering incorrect and unknown together). Junior (pre-med, 1st and 2nd years) and senior (3rd, 4th and 5th years) students were compared with Pearson’s chi-squared test. Univariate and multivariate analysis were used to identify associations between the total knowledge score (defined as the sum of correct answers) and seven exploratory variables determined a priori: age, gender, study years, exposition to organ donation in personal life, previous exposition to organ donation in medical school, and “would feel comfortable to answer questions of patients about OD.” This last variable was chosen by a panel of students because it was modifiable and because they hypothesized that lower knowledge could make students less confident, and therefore less engaged about organ donation in their practice. Univariate analysis was performed with Spearman’s rank correlation, excepted for gender, for which Student T-test for independent groups was used. Questionnaires containing less than 70% of answers or missing demographic data were not included. Comparative demographic data were provided by each faculty of medicine.

## Results

From 3344 medical students, 793 answered the questionnaire, of which 43 were excluded because they had completed less than 70% of the survey. Therefore, 750 respondents (22%) were included in analyses. Compared to the target population, there were more women while age and study year appeared similar (Table 1).

**Table 1:** Characteristics of respondents and target population

		All students	Junior students			Senior students		
			Pre-med	1st year	2nd year	3rd year	4th year	5th year
Respondents	Number of respondents n (%)	750 (100)	54 (7.2)	200 (26.7)	191 (25.5)	134 (17.9)	140 (18.7)	31 (4.1)
	Female n (%)	541 (72.1)	41 (75.9)	142 (71.0)	138 (72.3)	100 (74.6)	95 (67.9)	25 (80.6)
	Age Mean (SD)	22.5 (3.22)	20.0 (2.54)	21.5 (2.92)	22.5 (3.45)	22.6 (2.35)	24.3 (3.26)	24.5 (2.29)
*Target population (students from 3 French-speaking medical schools in Quebec)*	*Number of students* *n (%)*	*3344 (100)*	*217* *(6.5)*	*736 (22.0)*	*744 (22.2)*	*724 (21.7)*	*704 (21.1)*	*219 (6.5)*
	*Female* *n (%)*	*2175 (65.0)*	*Not available*					
	*Age* *Mean*	*21.8–24.4*^a^	*Not available*					

^a^Mean age varies between 21.8 years old in one medical school to 24.4 years old in another medical school

### Knowledge about organ donation

Table 2 presents the frequency of correct answers to questions about knowledge.

**Table 2:** Frequency of correct answers regarding knowledge about organ donation

Questions	Frequency of correct answers			*p-*value
	All students *n* = 750 (%)	Junior^**a**^ *n* = 445 (%)	Senior ^**a**^ *n* = 305 (%)	
Is it possible for someone who is brain-dead to recover and come back to life? *Yes; No (correct)*	679 (90.5)	391 (87.9)	288 (94.4)	**0.003**
Is it possible to say that a person is actually dead if he is brain-dead, but his heart is still beating? *Yes (correct); No*	571 (76.1)	348 (78.2)	223 (73.1)	0.108
By what means can one legally give consent to organ donation in Quebec?				
*Sign the sticker on the health insurance card (correct)*	685 (91.3)	413 (92.8)	272 (89.2)	0.083
*Sign the sticker on the driver’s licence (false)*	653 (87.1)	379 (85.2)	274 (89.8)	0.061
*Sign the hospital’s registry (false)*	627 (83.6)	363 (81.6)	264 (86.6)	0.070
*Register with the health insurance registry (correct)*	437 (58.3)	268 (60.2)	169 (55.4)	0.189
*Register with the Quebec notaries’ registry (correct)*	298 (39.7)	196 (44.0)	102 (33.4)	**0.004**
In Quebec, the family members of the donor have the last word when it comes to consent to organ donation. *True (correct); False*	536 (71.5)	336 (75.5)	200 (65.6)	**0.003**
In Quebec, which organization is responsible for the management of organ donations? *Héma-Quebec; Greffe Québec; Transplant Quebec (correct); Canadian Blood Services; I don’t know*	499 (66.5)	295 (66.3)	204 (66.9)	0.866
Many organs can be grafted in Quebec in 2017. Among the following lists, which is correct? *Heart, lungs, pancreas (correct); Kidney, liver, uterus; Intestines, heart, bladder; I don’t know,*	446 (59.5).	264 (59.3)	182 (59.7)	0.924
To proceed with an organ donation, is it required that the donor be brain-dead? *Yes/No (correct; donation after circulatory death)*	234 (31.2)	137 (30.8)	97 (31.8)	0.768

Two additional questions are described in the text and presented in Additional file.

^a^*Junior students include pre-med, 1st year and 2nd year students, before clinical rotations. Senior students include students that have started their clinical rotations,* i.e. *3rd to 5th year students*

Overall, almost all students (*n* = 679; 91%) knew that neurological death is irreversible. Three-quarter (*n* = 571; 76%) recognized that a patient could be neurologically deceased even if his heart is still beating, while one quarter did not. Two third (*n* = 499, 67%) correctly identified the provincial organization responsible for organ donation. The majority (*n* = 446, 60%) correctly identified that “heart, lung and pancreas” could be transplanted in Quebec. The majority (*n* = 516, 69%) were not aware of the option of organ donation after circulatory death. While almost all students (*n* = 685, 91%) knew that people could consent to organ donation by signing a sticker on their health insurance card, fewer students were aware of the two organ donation registries available in the province (respectively *n* = 437, 58% and *n* = 298, 40%).

Exposure to organ donation during medical studies and comfort to answer patients’ questions about organ donation were associated with better knowledge (Table 3). Importantly, the level of knowledge did not increase throughout the medical studies.

**Table 3:** Factors associated with better knowledge

	Univariate analysis	Multivariate linear regression	
	*p-*value	Standardized coefficients	*p-*value
Age	0.072	−0.033	0.383
Gender	0.428	0.062	0.077
Study year	0.934	0.014	0.727
Exposition to OD in personal life	**< 0.001**	0.056	0.127
Exposition to OD during medical studies	**< 0.001**	0.151	**< 0,001**
Would feel comfortable to answer questions from patients about OD	**< 0.001**	0.199	**< 0,001**

Some questions measured knowledge but also reflect perception about organ donation (Additional file, Table A3): almost all respondents (*n* = 659, 88%) were aware of the organ shortage in Canada, but less than half (*n* = 317, 42%) agreed that the cost of a renal transplant was lower than the treatment of a patient with end-stage renal insufficiency.

### Attitudes toward organ donation and potential determinants

Table 4 describes the frequency of respondents that chose each answer of various questions evaluating attitude about organ donation, that was generally positive.

**Table 4:** Frequency of answers concerning attitude toward organ donation

Questions	**Completely disagree** n (%)	**Somewhat disagree** n (%)	**Neutral** n (%)	**Somewhat agree** n (%)	**Completely agree** n (%)
Upon my death, I wish to donate my organs	4 (0.5)	10 (1.3)	15 (2.0)	76 (10.1)	645 (86.0)
I would donate a kidney to a loved one at some point in my lifetime	4 (0.5)	29 (3.9)	147 (19.6)	309 (41.2)	261 (34.8)
Learning activities pertaining to organ donation should be a component of undergraduate medical education	3 (0.4)	9 (1.2)	49 (6.5)	689 (91.9) ^a^	
I would feel comfortable answering a patient’s questions about organ donation	34 (4.5)	203 (27.0)	164 (21.9)	247 (32.9)	102 (13.6)
Family members of organ donors are comfortable discussing the process of organ donation	22 (2.9)	270 (36.0)	305 (40.7)	126 (16.8)	27 (3.6)
Organ donation is important for my training program	3 (0.4)	13 (1.7)	77 (10.3)	318 (42.4)	339 (45.2)
OD should be part of the preventive advice given by a family physician to an adult in good health	7 (0.9)	13 (1.7)	57 (7.6)	272 (36.3)	401 (53.5)
Only specialists who are familiar with OD (for example, an intensivist) should be tasked with recognizing a patient who meets the requirements for OD	189 (25.2)	299 (39.9)	136 (18.1)	93 (12.4)	33 (4.4)
Questions	**Definitely not** n (%)	**Unlikely** n (%)	**Uncertain** n (%)	**Likely** n (%)	**Definitely** n (%)
In the appropriate context, to what extent would you take responsibility for the following tasks in your role as a physician:					
*Consider if patients could be potential donors*	7 (0.9)	36 (4.8)	109 (14.5)	344 (45.9)	254 (33.9)
*Refer a potential donor*	7 (0.9)	27 (3.6)	108 (14.4)	310 (41.3)	298 (39.7)
*Discuss OD with the family of a potential donor*	7 (0.9)	46 (6.1)	152 (20.3)	335 (44.7)	210 (28.0)
Questions	**Never** n (%)	**1 time** n (%)	**2 times** n (%)	**3 times** n (%)	**4 times or more** n (%)
Throughout your medical studies, how many times have you been exposed to organ donation (courses, special activities, patient encounters)?	94 (12.5)	220 (29.3)	228 (30.4)	121 (16.1)	87 (11.6)
Questions	**Never** n (%)	**Rarely** n (%)	**On occasion** n (%)	**Often** n (%)	
In your personal life (outside of your studies), to what extent do you consider yourself to have been exposed to organ donation?	131 (17.5)	338 (45.1)	238 (31.7)	43 (5.7)	

^a^These two choices are combined because of low test-retest reliability between them. Both choices were kept in the questionnaire to maintain uniformity

The majority of respondents had legally given consent to organ donation after death (*n* = 613/750; 82%). Most were rarely (*n* = 338, 45%) or occasionally (*n* = 238, 32%) exposed to organ donation in their personal lives. Almost all (*n* = 656, 87%) were exposed at least once to organ donation during their medical training. Many respondents (*n* = 292, 39%) felt that family members of patients are not comfortable to discuss the process of organ donation or answered “neutral” to this question (*n* = 305, 41%).

### Need for training in organ donation

Most students (*n* = 649, 87%) felt the need to receive more training on organ donation. Half of them (*n* = 307/623, 49%) believed that the best time for additional training would be during clerkship, while the other half prefers the years before clerkship (*n* = 289/623, 46%) or the pre-medicine year (*n* = 27/623, 4.3%).

## Discussion

To our knowledge, this survey is the first to report knowledge and attitude about organ donation among medical students from Quebec’s universities, or among more than one university in Canada. Although respondents came from one province and are French-speaking, medical training should be comparable between provinces in Canada, because of its national regulating body. This could allow some generalization to other provinces, at least to raise potential educational needs. Furthermore, findings seem consistent with previous surveys elsewhere. Poor understanding of brain death was not rare, as observed in a survey of Ohio medical students: 73% did not agree that a “brain-dead person is legally dead.” [13] This limited understanding may compromise adequate explanation for families, but it may be remediated during residency. Most students were unaware of organ donation after circulatory death even if it represents a promising way to increase organ donation in Canada [10]. Interestingly, knowledge of senior medical students was not better than their junior colleagues. Similarly, in a survey conducted at Queen’s medical school (Ontario), the mean number of correct answers only slightly improved with study years [17]. Items in our survey may not be taught in medical school, but it can also suggest a lack of training about organ donation. Otherwise, findings suggest that medical students are strongly in favour of organ donation, as observed in other studies [13]. Compared to the Canadian general population, respondents were more likely to consent to organ donation: for example, only 51% of Canadians had already made the decision to donate their organs at the time of their death, even if 95% were supporting organ donation [20]. Medical students may be more sensitive to people waiting for transplantation, but it can also indicate non-response bias. Many respondents felt that families would not be comfortable to discuss organ donation. In contrast, a survey found that the majority of Canadians (69%) think that “It should be mandatory that family of critically ill patients be approached by healthcare providers to tell them about their options regarding organ donation.” [20] Students could anticipate negative reactions, and therefore be reluctant to engage in discussion, so they should be prepared adequately. Fortunately, half of respondents would feel comfortable to answer patients’ questions about organ donation, despite some knowledge gap. This question could have been understood in two different ways: feeling confident about its own capacity to answer patients’ questions or feeling that it is appropriate for a future doctor to answer their questions. This item was significantly associated with total knowledge in univariate and multivariate models.

Indeed, respondents expressed need for more training. Since students have a favourable attitude toward organ donation, educational interventions could focus on the recognition of potential neurologically or circulatory deceased donors, and more practical aspects (e.g., steps and actions to do in this situation; how to approach families about organ donation; legal aspects of consent). For example, students could determine whether fictional patients would be eligible for organ donation. Videos or simulations could develop the knowledge and the interpersonal skills required to discuss with patients and families about organ donation and to answer their questions. For instance, students could watch a recorded discussion between a trained physician and a family, and then simulate with peers.

## Limitations

The risk of non-response bias can be increased by the participation rate of 22%: it is plausible that students who chose to participate were more interested in organ donation, and possibly more knowledgeable. Knowledge and attitude would be overestimated. The participation rate is higher than in the National Physicians Survey from Canadian Medical Association (8.5%) but lower than in the wellbeing survey from the Canadian Medical Student Federation (40%) [21, 22]. Desirability bias could also overestimate attitude, despite anonymity. Differential recall bias could overestimate the association between knowledge and past exposure to organ donation, since more favourable and knowledgeable students might better remember their exposure. Results may provide insight but may not be fully generalized to other Canadian medical schools, considering different cultures, medical teaching and organ donation systems.

## Conclusion

Fortunately, future physicians who answered this survey seem strongly favourable to organ donation, both personally and professionally. Yet they have limited knowledge about brain death and organ donation in the Canadian context. The lack of improvement with study years may suggest the need for a formal curriculum on organ donation and transplantation, as expressed by respondents.

## Supplementary Information

**Additional file 1.** This additional file contains three tables as indicated in the Methods’ and Results’ sections: comparison with the theory of planned behaviour (Table A1); full questions and test-retest reliability of the questionnaire (Table A2); and frequency of correct answers about knowledge, not presented in Table 2 (Table A3).

## Data Availability

The datasets used and/or analyzed during the current study are available from the corresponding author on reasonable request.

## Abbreviations

OD: Organ donation
SD: Standard deviation
