# Supplementary material for: Attitude and knowledge of medical students about organ donation – training needs identified from a Canadian survey
Source: BMC Med Educ. 2021 Jul 5;21:368. doi: 10.1186/s12909-021-02736-2 (PMC8258931; doi:10.1186/s12909-021-02736-2)
Supplement: Supplementary file 1 — Additional file 1. This additional file contains three tables as indicated in the Methods’ and Results’ sections: comparison with the theory of planned behaviour (Table A1); full questions and test-retest reliability of the questionnaire (Table A2); and frequency of correct answers about knowledge, not presented in Table 2 (Table A3). [file 12909_2021_2736_MOESM1_ESM.docx]

**Additional File**

**Attitude and Knowledge of Medical Students about Organ Donation – Training Needs Identified from a Canadian Survey**

**Authors:**

Philippe Robert^1,2^

Félix Bégin^1^

Sasha Ménard-Castonguay^1^

Anne-Julie Frenette^3^

Hector Quiroz-Martinez^1^

François Lamontagne^1^

Émilie-Prudence Belley-Côté^4^

Frédérick D’Aragon^1^

^1^Université de Sherbrooke, Faculté de médecine et des sciences de la santé; 3001, 12e Avenue Nord, Sherbrooke, QC, Canada, J1H 5N4

^2^Université Laval, Faculté de médecine; 1050, Avenue de la Médecine, Québec, QC, Canada G1V 0A6

^3^Université de Montréal, Faculté de pharmacie; 2940, Chemin de Polytechnique, Montréal, QC, Canada, H3T 1J4

^4^McMaster University, Faculty of Health Sciences; 1200 Main Street West, Hamilton, ON, Canada, L8N 3Z5

**Corresponding Author:**

Philippe Robert : [philippe.robert.2@ulaval.ca](mailto:philippe.robert.2@ulaval.ca)

This additional file presents detailed data about questions that were not presented in the main results of the article and data about the validity analysis of the questionnaire.

**Table A1: Comparison with the theory of planned behaviour**

| Concept | Adaptation to organ donation | Questions |
| --- | --- | --- |
| **Behaviour** | Participating to the organ donation system as a physician | Not evaluated. |
| **Intention** | Intention to participate to the organ donation system as a physician | *In the appropriate context, to what extent would you take responsibility for the following tasks in your role as a physician:*  Consider if patients could be potential donors;  Refer a potential donor;  Discuss OD with the family of a potential donor |
| **Attitude** | Attitude toward organ donation and toward the role of the physician in the organ donation system | Have you legally given consent to donate your organs?  Upon my death, I wish to donate my organs.  I would donate a kidney to a loved one at some point in my lifetime.  Learning activities pertaining to organ donation should  be a component of undergraduate medical education.  OD should be part of the preventative advice given by a family physician to an adult in good health.  Only specialists who are familiar with OD (for example, an intensivist) should be tasked with recognizing a  patient who meets the requirements for OD |
| **Subjective norm** | Perception of social and professional pressure to participate in organ  donation system | Organ donation is important for my training program. |
| **Perceived behavioural**  **control** | Perception about how easy or difficult  it is to participate in the organ  donation system | I would feel comfortable answering a patient’s questions about organ donation.  Family members of organ donors are comfortable discussing the process of organ donation.  Do you feel the need to receive additional training on organ donation? |
| **Potential determinants (not in Planned Behaviour Theory)** | | In your personal life (outside of your studies), to what extent do you consider yourself to have been exposed to organ donation?  Throughout your medical studies, how many times have you been exposed to organ donation (courses, special activities, patient encounters)? |

**Table A2: Full questions and Test-retest reliability of the questionnaire**

| Items | Questions | | Test-retest reliability  Cohen’s kappa (quadratic-weighted) |
| --- | --- | --- | --- |
| Knowledge | | | |
| Neurological determination of death: irreversibility | Is it possible for someone who is brain-dead to recover and come back to life? *Yes/No* | | 1.00 |
| Neurological determination of death: no circulatory criteria | Is it possible to say that a person is actually dead if he is brain-dead, but his heart is still beating? *Yes/No* | | 1.00 |
| Means to express OD consent | By what means can one legally give consent to organ donation in Quebec? | *Sign the sticker on the health insurance card* | 1.00 |
|  |  | *Register with the RAMQ registry* | 0 (1/10 discordance)* |
|  |  | *Sign the sticker on the driver’s licence (false)* | 1.00 |
|  |  | *Sign the hospital’s registry (false)* | 0 (2/10 discordance)* |
|  |  | *Register with the Quebec notaries’ registry* | 0 (1/10 discordance)* |
| Consent to OD: practical role of family | In Quebec, the family members of the donor have the last word when it comes to consent to organ donation. *True/False* | | 0.63 |
| Name of Quebec’s organ transplantation organism | In Quebec, which organization is responsible for the management of organ donations? *Five choices* | | 1.00 |
| Organs appropriate for transplantation | Many organs can be grafted in Quebec in 2017. Among the following lists, which is correct? *Three choices* | | 0.80 |
| Recognize that there is another way to proceed to OD than neurological determination (circulatory determination) | To proceed with an organ donation, is it required that the donor be brain-dead? *Yes/No* | | 0.82 |
| Awareness of organ shortage | What is the current situation in Quebec with respect to the need and the supply of organs for transplantation | | 1.00 |
| Cost-beneficence of kidney transplantation | The costs of kidney transplantation are less than the benefits to society | | 0.69 |
| Attitude | | | |
| Legally consent to OD | Have you legally given consent to donate your organs? | | All yes |
| Wish to donate his organs | Upon my death, I wish to donate my organs | | 1.00 |
| Would consent to living kidney donation | I would donate a kidney to a loved one at some point in my lifetime | | 1.00 |
| Agree with OD training in medical school | Learning activities pertaining to organ donation should be a component of undergraduate medical education | | 0 ** |
| Exposition to OD in personal life | In your personal life (outside of your studies), to what extent do you consider yourself to have been exposed to organ donation? | | 0.66 |
| Exposition to OD during medical studies | Throughout your medical studies, how many times have you been exposed to organ donation (courses, special activities, patient encounters)? | | 1.00 |
| Would feel comfortable to answer questions of patients about OD | I would feel comfortable answering a patient’s questions about organ donation | | 0.78 |
| Family members of organ donors are comfortable to discuss the process of OD | Family members of organ donors are comfortable discussing the process of organ donation | | 0.86 |
| OD is important for its training program | Organ donation is important for my training program | | *Item added after validation.* |
| Need additional training on OD | Do you feel the need to receive additional training on organ donation? | | 1.00 |

* Since the question was binary, a few discordances resulted in poor Cohen’s kappa.

** Cohen’s kappa was very poor because many students answered, “Somewhat agree” instead of “Strongly agree” and *vice versa*. It was decided to keep the same wording for the choices of answers, to make it easier to complete the questionnaire, but to consider “Somewhat agree” and “Strongly agree” together.

**Table A3: Frequency of correct answers about knowledge, not presented in Table 2**

|  | All students  n = 750 (%) | Junior*  n = 445 (%) | Senior *  n =305 (%) | p-value |
| --- | --- | --- | --- | --- |
| What is the current situation in Quebec with respect to the need for organ donation and the supply of organs for donation?  *The supply greatly surpasses the demand;*  *The supply slightly surpasses the demand);*  *They are equivalent;*  *The demand slightly surpasses the supply;*  *The demand greatly surpasses the supply (correct).* | 659 (87.9) | 392 (88.1) | 267 (87.5) | 0.821 |
| The costs associated with a kidney transplant are less than the benefits that it provides to society.  *Completely agree (correct); Somewhat agree (correct);*  *Neutral; Somewhat disagree; Completely disagree.* | 317 (42.3) | 191 (42.9) | 126 (41.3) | 0.661 |

**Junior students include pre-med, 1^st^ year and 2^nd^ year students, before clinical rotations. Seniors students include students that have started their clinical rotations, i.e. 3^rd^ to 5^th^ year students.*
